# Revision of the fern genus *Orthiopteris* (Saccolomataceae) in Malesia and adjacent regions

**DOI:** 10.3897/phytokeys.53.4955

**Published:** 2015-07-21

**Authors:** Thien Tam Luong, Peter H. Hovenkamp, Marc S. M. Sosef

**Affiliations:** 1 Department of Ecology - Evolutionary Biology, Viet Nam National University Ho Chi Minh city (VNUHCM) - University of Science. 227 Nguyen Van Cu, Ho Chi Minh City, Vietnam; 2 Naturalis Biodiversity Center, section Botany. PO Box 9517, 2300 RA Leiden, The Netherlands; 3 Botanic Garden Meise, Nieuwelaan 38, 1860 Meise, Belgium

**Keywords:** *Orthiopteris*, taxonomy, new species, new varieties, Malesia, Madagascar, Polynesia, Melanesia, Saccolomataceae

## Abstract

A taxonomic revision of the Old-World representatives of the fern genus *Orthiopteris* is presented. We recognize eight species, one of which is newly described (*Orthiopteris samoensis*), and five varieties, of which two are newly described (Orthiopteris campylura var. insularis and Orthiopteris campylura var. laxa). *Orthiopteris acuminata*, *Orthiopteris caudata*, *Orthiopteris minor* and *Orthiopteris kingii* are all reduced to varieties of *Orthiopteris campylura*.

## Introduction

The genus *Orthiopteris* was first recognized by Copeland (1929a) to accommodate *Davallia ferulacea* Moore (= *Orthiopteris ferulacea* (Moore) Copel.) from Fiji. Later that year, Copeland (1929b) described the genus *Ithycaulon* Copel. to accommodate the species *Ithycaulon moluccanum* (Blume) Copel. (= *Davallia moluccana* Blume.), *Ithycaulon caudatum* (Copel.) Copel., and *Ithycaulon inaequale* (Kunze) Copel., noting an affinity between *Ithycaulon* and *Saccoloma* Kaulf. However, in 1947, he (Copeland 1947) merged *Ithycaulon* with *Orthiopteris* (with *Orthiopteris* having priority), while maintaining the idea that *Orthiopteris* and *Saccoloma* are “near relatives”.

Copeland (1929b) considered *Ithycaulon* to be close to *Dennstaedtia* based on similarities in frond dissection and sori. In his Genera Filicum (1947) he arranged *Orthiopteris* next to *Dennstaedtia*. He was followed by most pteridologists (Holttum 1947; Jaman and Latiff. 1998; Kato 1996). Kramer (1990) sunk *Orthiopteris* into *Saccoloma*, a genus in the subfamily *Saccolomatoideae* of the *Dennstaedtiaceae*. Molecular phylogenetic studies ambiguously support this classification. Wolf (1995) placed *Saccoloma* in a group with mainly Pteridoid affinities, whereas others resolved *Saccoloma* at the base of the Polypod lineage (Pryer et al. 2004) or in a position more closely related to Lindsaeoid than to Dennstaedtioid ferns (Schuettpelz and Pryer 2007). Recent classifications treat *Orthiopteris* as one of the two genera within the family *Saccolomataceae* (Smith et al. 2006; Christenhusz et al. 2011). The description of the family *Saccolomataceae* Doweld was validly published only recently (Doweld and Reveal 2008).

The question whether *Orthiopteris* is distinct from *Saccoloma* within the *Saccolomataceae* is also controversial, and requires further study (Smith et al. 2006; Christenhusz et al. 2011). Many authors have treated *Orthiopteris* as a synonym of *Saccoloma* (Tryon and Tryon 1982b; Kramer et al. 1990; Parris et al. 1992; Smith et al. 2006). Both genera share a similar rhizome anatomical structure, sorus shape and spore morphology. In transverse section the rhizome of *Orthiopteris* spp. and *Saccoloma elegans* Kaulf. shows two concentric, somewhat discontinuous, rings of vascular bundles. Both genera have pouch-shaped sori and tetrahedral-globose, with a plain exospore and perispore with low branching ridges. However, *Orthiopteris* fronds are bipinnate or more decompound, unlike the simple-pinnate fronds of the type species of *Saccoloma*, *Saccoloma elegans* (Kramer 1990). Using a single *rbcL* gene phylogeny, Wolf (1995) found *Orthiopteris* as here circumscribed in two widely different clades. He reported *Saccoloma moluccanum* (based on a specimen from the Philippines, and therefore most likely corresponding with Orthiopteris campylura var. campylura) in a clade with (American) *Saccoloma elegans*, and *Orthiopteris kingii* (based on a specimen from Indonesia) in a far more basal position. Since then there has not been any other phylogenetic study including representatives of both *Orthiopteris* and *Saccoloma*. Subsequent studies including only *Saccoloma* (Pryer et al. 2004; Schuettpelz and Pryer 2007; Rai and Graham 2010; Lehtonen et al. 2012) all place *Saccoloma* in a basal position, although with some uncertainty regarding the exact position (Lehtonen et al. 2012). We performed a reanalysis (not reported) of the *rbcL* marker of all accessions used in these studies, which placed all these in a clade with the *Orthiopteris* accession that Wolf (1995) placed in a basal position. A more densely sampled analysis is clearly needed to resolve the exact relationships between the two genera and their phylogenetic position.

## Scope of this study

In the Malay-Pacific region, 11 *Orthopteris* species have been reported: *Orthiopteris acuminata* (Rosenst.) Copel, *Orthiopteris campylura*, *Orthiopteris caudata* (Copel.) Copel., *Orthiopteris cicutarioides* (Baker) Copel., *Orthiopteris ferulacea*, *Orthiopteris firma* (Kuhn) Brownlie, *Orthiopteris henriettae*, *Orthiopteris kingii* (Bedd.) Holttum, *Orthiopteris minor* (Hook.) Copel., *Orthiopteris tenuis* (Brack.) Brownlie, and *Orthiopteris trichophylla* Copel. (Copeland 1950; Holttum 1954; Copeland 1958; Brownlie 1969; Parris 1992; Beaman et al. 1992; Whistler 1994; Kato 1996; Brownsey and Perrie 2011). There is, however, uncertainty about the taxonomic status and nomenclature of some of these names. Here, we attempt to elucidate the specific boundaries and geographic distributions of all taxa occurring in this region by revising the available material. We also include the Malagasy endemic species *Orthiopteris henriettae* Baker (Tardieu-Blot 1958), to assess the status of this geographically isolated taxon. We hope this study will provide an impetus for a worldwide revision of *Orthiopteris* and *Saccoloma*, including also the American species *Orthiopteris domingensis* (Spreng.) Copel., *Orthiopteris inaequalis* (Kunze) Copel., and *Saccoloma elegans*.

## Material and methods

This study was based entirely on herbarium specimens. In total, 240 specimens have been examined from K, L, MICH, MO, NY, SING and additional images or on-line images provided by BM, K, MICH, P, and the JSTOR Global Plants database (plants.jstor.org). Herbarium abbreviations follow Index Herbariorum (Thiers continuously updated). Sheets seen as digital image only are marked with “*”. Data of all the studied specimens were entered into the BRAHMS database at L for storage and further analysis of geographic distribution. All studied specimens are listed in Appendix. We have selected lectotypes whenever necessary to resolve ambiguities in the application of a name. Most of the morphological characters were examined and measured with a stereo microscope or a compound microscope. In addition to light microscopy, scanning electron microscopy (using a Jeol JSM 7600F FEG-SEM) was used to study structure and ornamentation of the spores.

### Morphology

*Orthiopteris* is often a large-sized fern; with a stout, erect rhizome and fronds often more than a meter long. Herbarium collections are often incomplete, and this makes an evaluation of the differentiating characters difficult. For the distinctions between the species, we have studied the following characters.

### Rhizome

Due to lack of complete specimens, a thorough assessment of the variability of the rhizome morphology was impossible. From the available material, rhizomes can be assessed to be uniformly erect, and radially organized, and may form aerial trunks exceptionally to 1 m high. Characteristically, the vascular system shows two concentric cylinders of meristeles (well-illustrated in Bower 1918; 1923). The morphology of the rhizome scales appears to show very little variation, and we have not found it useful for species distinction. In most of the species, rhizome scales are multistratose and pseudopeltate, tapering to a long narrow acumen from a rounded-cordate base, the margin usually being eroded and unistratose (Figure 4).

### Frond divisions

**Figure 1.**
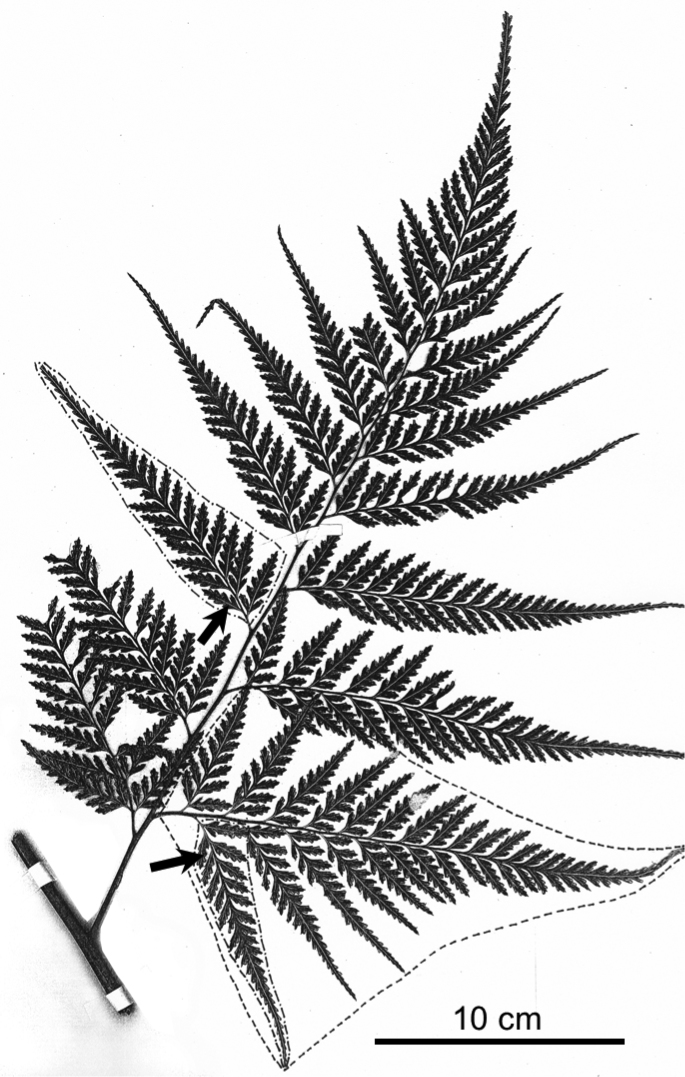
Basal pinna of *Orthiopteris firma*, showing quadripinnatifid dissection and terminology. bipinnate segment 2^nd^ order. isomorphic segments 2^nd^ and 3^rd^ order, arrows: ultimate segments. Franc 335 (K).

Division and venation of fronds are among the most confusing characters in *Orthiopteris* as the fronds are dissected to many levels, in many specimens reaching to quadripinnate. The gradual decrease in dissection from base to apex produces an almost perfectly fractal pattern, where the most basal pinnules are almost perfectly isomorphic with more apical pinnae (Figure 1). This makes it difficult to judge this character in fragmentary specimens unless it is known exactly from which part of a frond the fragment is taken. In our species descriptions, we concentrate on the major (pinnae) and on the ultimate divisions (segments) (Figure 1).

**Figure 2.**
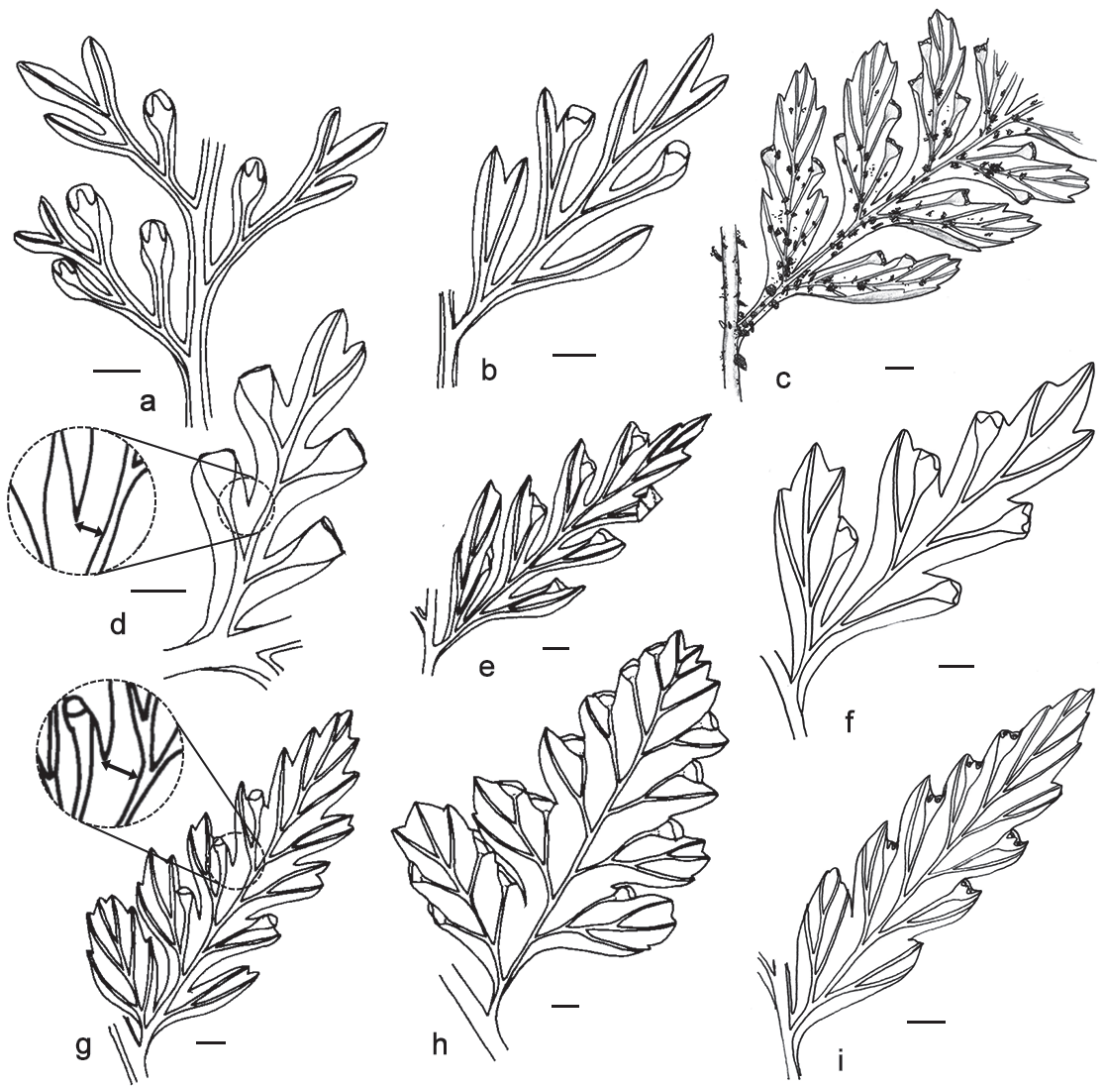
Ultimate segments. Arrows show distance from base of the shallowest sinuses to nearest costule/vein. **a** *Orthiopteris ferulacea*, Smith 8932 (K) **b** *Orthiopteris cicutarioides*, Carr 13257 (K) **c** *Orthiopteris samoensis*, Reinecke 97a (L) **d** *Orthiopteris trichophylla*, Brass 12239 (L) **e** Orthiopteris campylura var. cautada, Carr 12518 (L) **f** Orthiopteris campylura var. laxa Mueller s.n. (L) **g** Orthiopteris campylura var. kingii, Brass 12941 (MICH) **h** *Orthiopteris henriettae*, Lam & Meeuse 5892 (L) **i** Orthiopteris campylura var. insularis Braithwaite 4110 (L). All scale bars 1 mm.

We define “ultimate segment” as the smallest distal unit with a branched venation around which lamina is incised completely, leaving no or very little lamina around the vein at its base. Margins are described as shallowly incised, crenate, dentate, or deeply lobed, with the degree of dissection quantified, when necessary, by the distance from the base of the shallowest sinuses to nearest costule/vein (Figure 2).

### Sori

**Figure 3.**
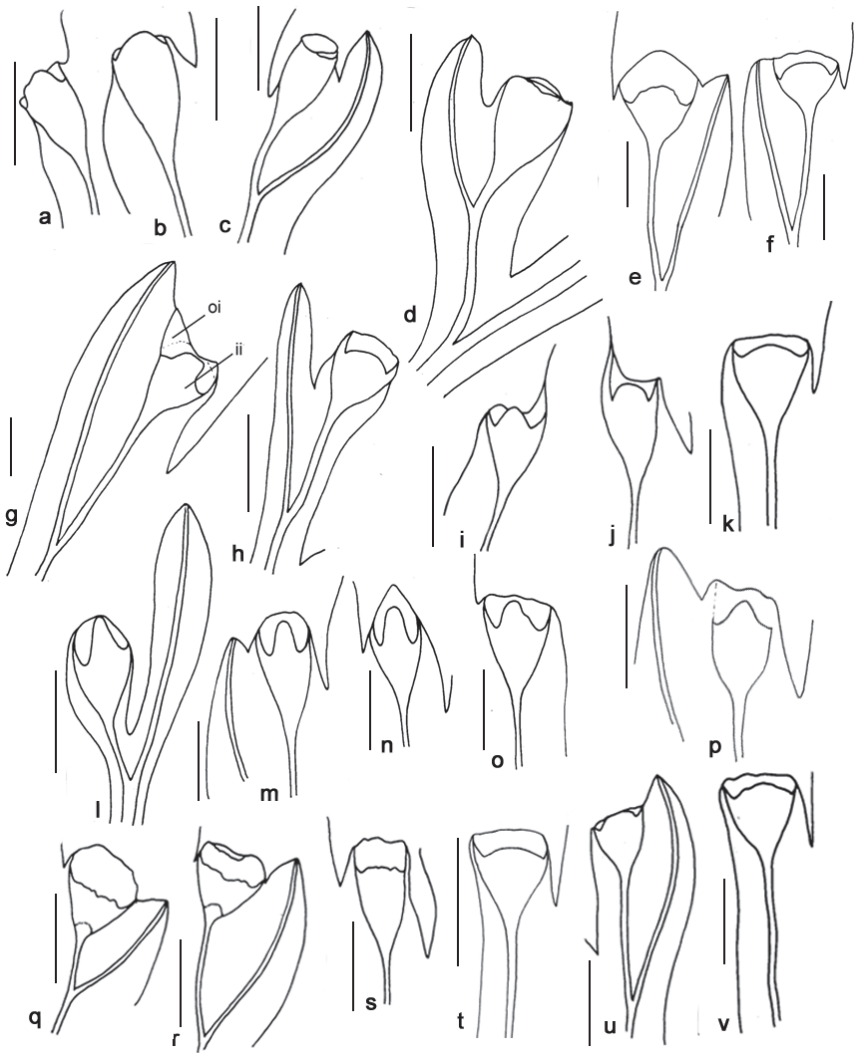
Sori of *Orthiopteris* seen from abaxial side. **a, b** Orthiopteris campylura var. campylura (Cuming 119, MICH) **c** Orthiopteris campylura var. kingii (Brass 12941, MICH) **d** *Orthiopteris trichophylla* (Brass 12239, L) **e** *Orthiopteris tenuis* (Smith 9154, L) **f** *Orthiopteris tenuis* (Smith 7274, L) **g** Orthiopteris campylura var. caudata (Carr 12518, L) **h** *Orthiopteris cicutarioides* (Carr 13257, K) **i, j** Orthiopteris campylura var. insularis (Braithwaite 4110, L) **k** *Orthiopteris tenuis* (small segment) (Degener 14648, L) **l** *Orthiopteris ferulacea* (Smith 8932, K) **m, n** *Orthiopteris firma* (Schlechter 14890, L) **o, p** Orthiopteris campylura var. laxa (Mueller s.n., L) **q, r** *Orthiopteris henriettae* (Lam & Meeuse 5892, L) **s** *Orthiopteris henriettae*. **t** *Orthiopteris samoensis* (Reinecke 97a, L) **u, v** *Orthiopteris samoensis* (Vaupel 312, L). All scale bars 1 mm, oi = outer indusium, ii = inner indusium.

The most variable and important characters for distinguishing *Orthiopteris* species are found in the sori, the inner indusium and the outer indusium. The inner indusium is a thin membrane on the abaxial surface of the lamina. The outer indusium is continuous with the lamina on the adaxial surface. Each sorus thus forms a pouch-shaped structure that contains up to 30 sporangia, but mostly less, that are immersed within the indusium when young, but variously exserted when mature. We distinguish and classify several soral shape types as shown in Table 1 (see also Figure 3).

**Table 1.** Different types of soral shapes.

Length of sorus Widest at	equal to width	longer than width
Mouth	Funnelform	Narrow funnelform
Above middle	Wide obovate	Narrow obovate
At middle	Round/Ovate	Elliptic

In addition, we use the following descriptive terms:

Outer indusium: (1) symmetric when it is not confluent with the margin but is abruptly set-off from the lamina margin on two sides (Figure 3a, b, c, d...); (2) asymmetric when it is confluent with the lamina margin on one side (Figure 3g, i, j...).

Inner indusium at apex: (1) obtuse-truncate without incision (Figure 3b, c, d,…), (2) extended into a lobe when it has a lobe 1/2–2/3 as wide as the entire sorus but shorter than 1/3 the length of the sorus (Figure 3g, h, i, j…), (3) extended into a narrow tongue, when it has a lobe less than ½ as wide as the entire sorus and longer than 1/3 the sorus length (Figure 3l, m, n, o…). To assess this character, it is necessary to examine more than one sorus because they can show some variations even on the same frond. In most species, the sori are in the same plane as the frond, but sori may also be curved towards the abaxial surface.

### Sporangia and spores

Characters of the sporangia are deemed not important for species distinction. Sporangia are stalked with a spherical to hemispherical capsule and a vertical, interrupted annulus with 16–24 indurated cells, which are more or less equal to distinctly unequal in size (Figure 5). Stalk and capsule are glabrous. All examined species have a more or less similar spore morphology resembling the American *Saccoloma elegans* (Tryon and Lugardon 1991: 269, fig. 90). Spores are trilete, tetrahedral-globose with a smooth or finely wrinkled exospore closely overlain with a perispore ornamented with ridges (Figure 6). The ridges strongly vary in width and density and in the degree to which they are sinuous and anastomosing.

**Figure 4.**
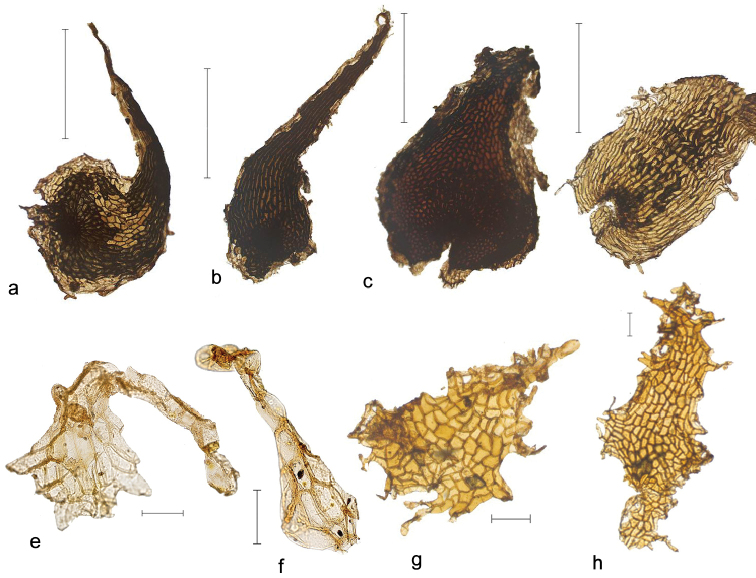
**a–d** Rhizome scales and **e–h** rachis scales of *Orthiopteris*. **a** *Orthiopteris cicutarioides* (Carr 13257, L) **b** *Orthiopteris ferulacea* (Smith 8932) **c** *Orthiopteris firma* (Green 1793, K) **d** Orthiopteris campylura var. kingi (Hoogland & Craven 10162, L) **e, f** Orthiopteris campylura var. kingi (Larhing 6910, L) **g, h** *Orthiopteris samoensis* (Reinecke 97a, L). Scale bars: 1 mm (**a–d**), 0.1 mm (**e–h**).

**Figure 5.**
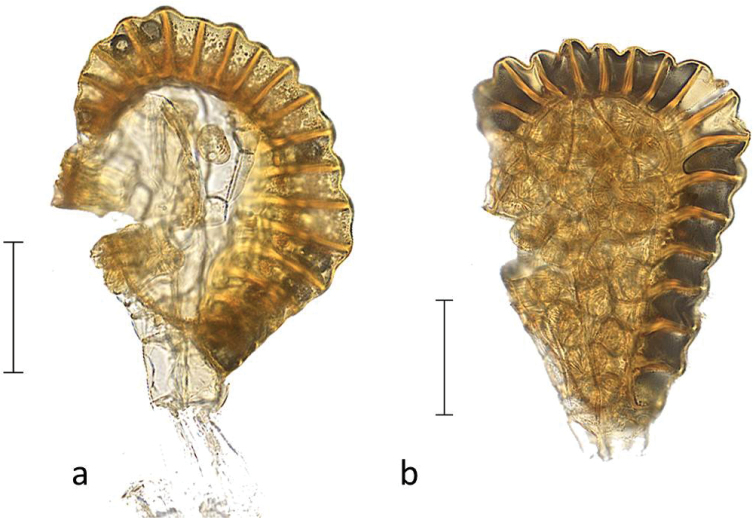
Sporangia of *Orthiopteris*, note the unequal-sized annulus cells. **a** *Orthiopteris henriettae* (Malcomber et al. 2246, MO) **b** *Orthiopteris firma* (Green 1793, K). All scale bar 0.1 mm.

**Figure 6.**
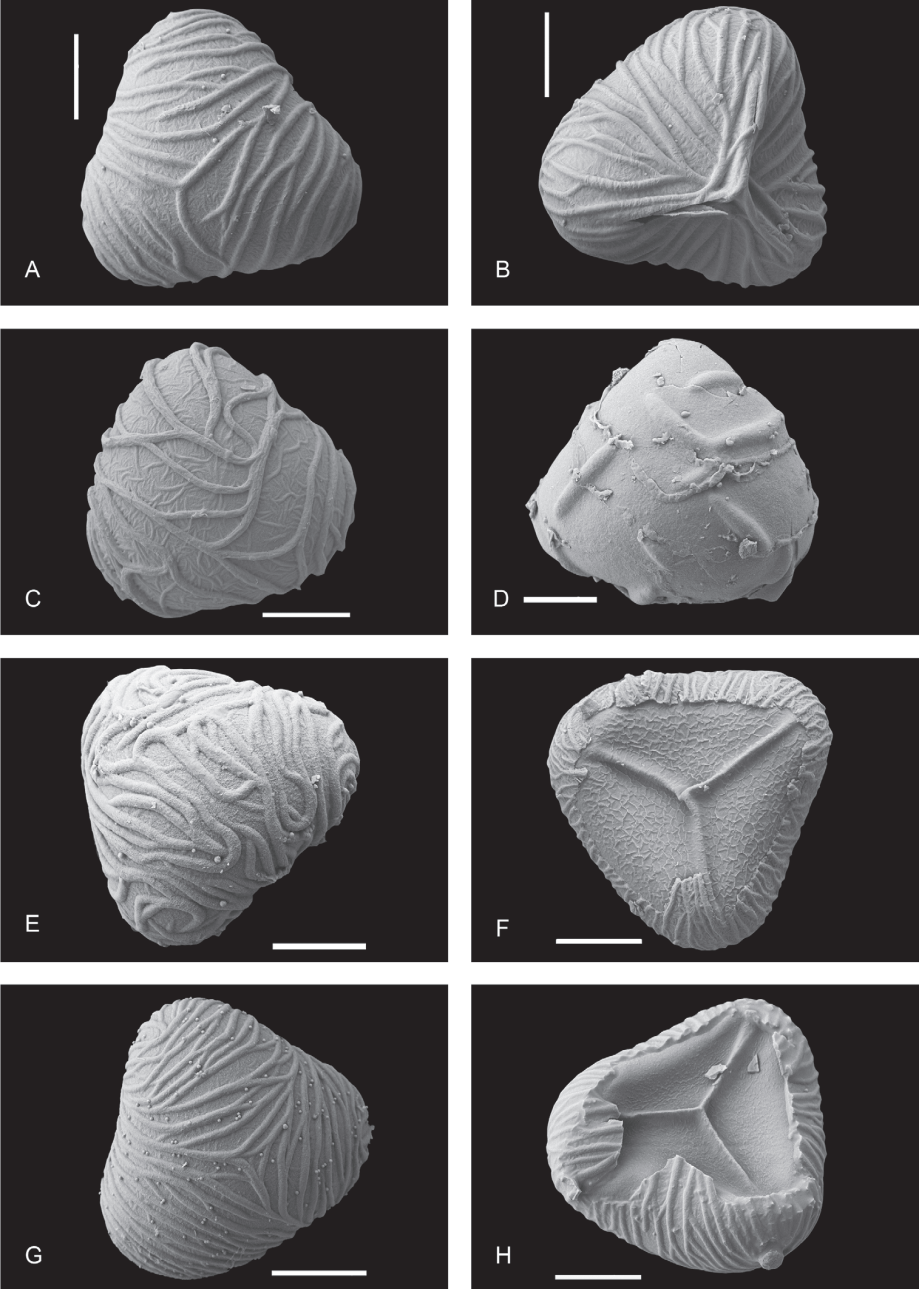
*Orthiopteris* spores. **a, b** *Orthiopteris firma* (Guillaumin & Baumann-Bodenheim 10318, L) **c** *Orthiopteris tenuis* (Smith 9154, L) **d** Orthiopteris campylura var. campylura (Elmer 11717, L) **e** Orthiopteris campylura var. caudata (Ridsdale 30654, L) **f** *Orthiopteris henriettae* (Lam & Meeuse 5892, L) **g, h** *Orthiopteris samoensis* (Sledge 1563, L). All scale bars 10 µm.

## Taxonomy

### Orthiopteris

Taxon classificationPlantaePolypodialesSaccolomataceae

Copel.

Orthiopteris Copel., Bernice P. Bishop Mus. Bull. 59: 14. 1929a. Type: *Davallia ferulacea* T. Moore = *Orthiopteris ferulacea* (T. Moore) Copel.
Ithycaulon auct. non Copel., Univ. Cal. Publ. Bot. 16: 79. 1929b. Type: *Davallia moluccana* Blume = *Ithycaulon moluccanum* (Blume) Copel.
Orthiopteris See also Excluded names.

#### Description.

Rhizome erect, stout, forming a trunk (2–)20–50(–100) cm high, in cross section showing two complete rings of vascular bundles, partly covered with scales, roots inserted on all sides of the rhizome especially in the older parts. Rhizome scales appressed at base, mostly basifix, pseudopeltate to peltate, mostly narrow lanceolate with long attenuate tips, thick, stiff, brittle, dark brown. Fronds tufted, erect, monomorphic. Stipes (10–)30–70(–90) cm long, 0.4–0.8 cm thick (at base), scaly or slightly roughened due to scale traces towards base, greenish-brown. Lamina tri- to quadripinnate, anadromous, deltoid or lanceolate, gradually attenuate towards apex, (30–)100–150(–200) × (30–)40–60(–100) cm, herbaceous to papyraceous, bright-green to brownish–green when dry, abaxial surface sometimes with scattered small scales. Segments alternating, the acroscopic sides of all divisions larger than basiscopic sides, except for the first basiscopic pinnules of the lowest two pinnae; ultimate segments sessile or very short stalked, trapeziform, asymmetric at base, shallowly to deeply lobed. Rachis and costae grooved on adaxial surface, veins dichotomously forked, free. Sori terminal on veins, apical or lateral on the lobe, marginal to sub-marginal, reflexed or not when dry, funnelform, obovate or elliptic, formed by an outer indusium continuous with the lamina and an inner indusium, affixed at sides and usually with same colour as the vein. Sporangia (3–)5–15(–30) per sorus, immersed within indusium when young, exserted when mature. Annulus cells 16–24, more or less equal to distinctly unequal in size. Spores tetrahedral-globose, 25–38 µm in polar view, 20–25 µm in lateral view; perispore surface ornamented with branching sinuous ridges, exospore smooth or weakly scabrate.

#### Geographical distribution.

**Figure 7.**
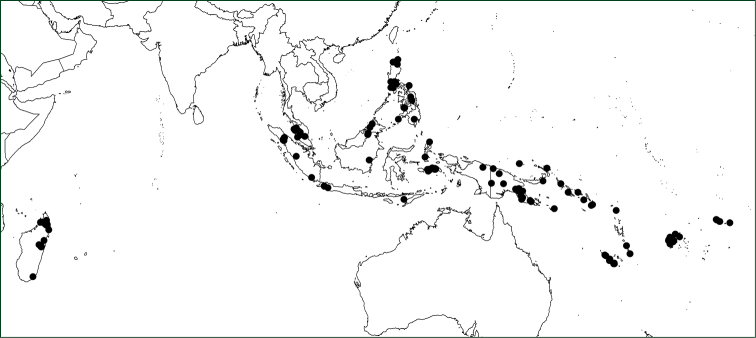
Distribution of *Orthiopteris* excl. *Saccoloma*.

**Figure 8.**
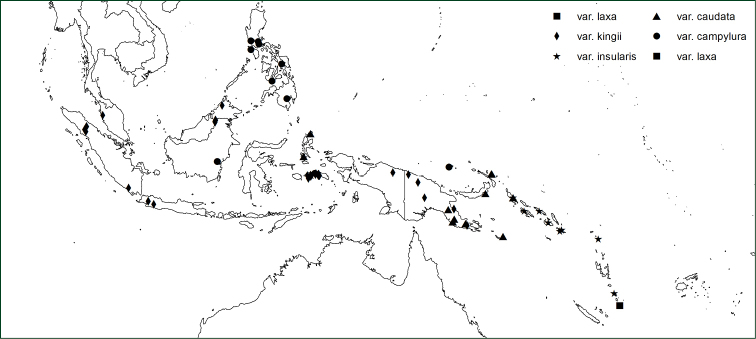
Distribution of *Orthiopteris campylura* and its varieties.

*Orthiopteris* has seven species occurring in the Malay-Pacific region, one of which is widespread in the area and consists of a number of geographically more or less weakly delimited varieties (Figure 7), the remaining six are more or less narrow endemics in the eastern part of the region (Figure 8). On Madagascar, there is a single species.

#### Key to the species

**Table:** 

1	Ultimate segments of fronds finely dissected, distance from base of sinuses to the nearest costule 0.1 mm or less; lobes usually with a single unbranched vein, or with 1–2 additional veins	**2**
–	Ultimate segments of fronds not finely dissected, distance from base of sinuses to the nearest costule more than 0.1 mm (usually more than 0.5 mm); lobes with veins pinnately branched into more than 3 veins	**5**
2	Ultimate segments completely dissected to the veins, thus forming a single lobe for each vein, lobe not narrowed toward base; sori equal to or wider than the sterile lamina part below its base	**3**
–	Ultimate segments not completely dissected to the veins, with 2–3 veins per lobe, lobe narrowed towards base; sori narrower than the sterile lamina part below its base	**4**
3	Sori elliptic-obovate, not reflexed when dried; inner indusium extending into a narrow tongue. Plant including rhizome not more than 1 m tall; stipes slender, 1–2 mm thick	**3. *Orthiopteris ferulacea***
–	Sori funnelform, reflexed when dried, inner indusium extending into a narrow tongue. Plant including rhizome more than 1 m tall; stipes thicker than 2 mm	**8. *Orthiopteris trichophylla***
4	Outer indusium asymmetric; inner indusium lobed with lobe less than 2/3 width of sorus. Angle between ultimate segment and costa usually larger than 40°	**2. *Orthiopteris cicutarioides***
–	Outer indusium symmetric; inner indusium obtuse, if lobed then lobe shallow and more than 2/3 width of sorus. Angle between ultimate segment and costa smaller than 40°	**7. *Orthiopteris tenuis***
5	Lamina margin with distinct yellow cartilaginous border; inner indusium extending into a narrow tongue about half the length of the entire sorus; sori elliptic to obovate	**4. *Orthiopteris firma***
–	Lamina margin without distinct yellow cartilaginous border; inner indusium not extending into a narrow tongue, but truncate or with a shallow, obtuse lobe (about 1/4–1/3 length of sorus); sori funnelform	**6**
6	Inner indusium distinctly shorter than outer, if almost equal then lamina thick and finely dissected	**7**
–	Inner indusium equal as or little shorter than outer, at least 2/3 as long; lamina thin and not finely dissected	**8**
7	Lamina 140–150 cm wide, as wide as long or wider, papyraceous, thin; ultimate segments stalked, wide trapeziform; veins darker than lamina; sori nearly as wide as long, fertile vein swollen at the joint with base of sori; inner indusium emarginate and irregularly eroded, not lobed	**5. *Orthiopteris henriettae***
–	Lamina 40–50 cm wide, longer than wide, herbaceous, not thin; ultimate segments sessile or very short stalked, narrow trapeziform; veins lighter than lamina; sori 2/3 as wide as long; fertile vein not swollen at the joint with base of sori; inner indusium entire, forming a shallow lobe.	**7. *Orthiopteris tenuis***
8	Abaxial surface of lamina with scattered irregular triangular scales; apex of inner indusium lobed and eroded. Sori situated on both acroscopic and basiscopic sides of lobes	**6. *Orthiopteris samoensis***
–	Abaxial surface of lamina without scales or with few hair-like scales; apex of inner indusium often entire and obtuse to truncate, if single-lobed then sori situated mostly on acroscopic side of lobes	**9**
9	Sori asymmetric; inner indusium with single lobe; lobe width less than 2/3 width of sorus	**10**
–	Sori symmetric, inner indusium obtuse to truncate, if forming a lobe then lobe width at least 2/3 width of sorus	**11**
10	Sori 0.6 mm or more wide	**12**
–	Sori not wider than 0.6 mm	**1d. Orthiopteris campylura var. insularis**
11	Sori strongly reflexed when dry	**1c. Orthiopteris campylura var. kingii**
–	Sori in the plane of the lamina when dry	**1a. Orthiopteris campylura var. campylura**
12	Lobes of ultimate segments blunt, obtuse; sori longer than wide; outer indusium not confluent with lamina margin on one side but incised	**1e. Orthiopteris campylura var. laxa**
–	Lobules of ultimate segments acute; sori almost as wide as long; outer indusium confluent with lamina margin on one side	**1b. Orthiopteris campylura var. caudata**

### 1. Orthiopteris campylura

Taxon classificationPlantaePolypodialesSaccolomataceae

(Kunze) Copel., Fern Fl. Philip. 1: 87. 1958.

Davallia inaequalis var. minor Hook., Sp. Fil. 1: 180, pl. 58A. 1846. Lectotype (selected here): PHILIPPINES. Luzon, *Cuming 119* (K, on 2 sheets: K000794851*, K000794853*, isolectotypes: K: K000794864*, MICH: MICH1259585*).
Davallia campylura Kunze, Bot. Zeitung (Berlin) 8: 132. 1850. Lectotype (selected here): PHILIPPINES. Luzon, *Cuming 119* (herb. Kunze, not traced, isolectotypes: K: K000794851*, K000794853*, K000794864*, MICH: MICH1259585*, see below).
Saccoloma campylurum (Kunze) Mett. Ann. Sci. Nat. Bot. sér. 4, 15: 80. 1861. Type. Based on *Davallia campylura* Kunze
Saccoloma moluccanum (Blume) Mett. in Kuhn var. stenolobum Christ, Bull. Herb. Boissier, ser. 2 6: 1005. 1906. Type. PHILIPPINES. Mabacal/Rizal, III, 1906, Loher s.n. (holo: P, on 4 sheets: P01566098*, P01566099*, P01566100*, P01566101*).
Saccoloma minus (Hook.) C.Chr., Gard. Bull. Straits Settlem. 4: 399. 1929. Type. Based on var. minor Hook.
Ithycaulon minus (Hook.) C.Chr., Index. Fil. Suppl. 3: 116. 1934. Type. Based on var. minor Hook.
Orthiopteris minor (Hook.) Copel., Gen. Fil.: 50. 1947. Type. Based on var. minor Hook.

#### Type.

PHILIPPINES. Luzon, *Cuming 119*

(K000794851 [http://specimens.kew.org/herbarium/K000794851],

K000794853 [http://specimens.kew.org/herbarium/K000794853],

K000794864 [http://specimens.kew.org/herbarium/K000794864];

MICH, 1259585 [http://quod.lib.umich.edu/h/herb2ic/x-mich1259585/mich1259585.tif]).

non *Saccoloma alatum* (Heward) (J. Sm.) Mett., Ann. Sci. Nat., Bot. sér. 4, 15: 80. 1861, nec *Microlepia alata* J. Sm., J. Bot. (Hooker) 3: 416. 1841, which is based on *Davallia alata* Heward, Mag. Nat. Hist. & J. Zool. II. ser. 2, 2: 465. 1838, nom. illeg, non Blume (1828).

Type. Jamaica, *Heward*. John Smith (1841) applied this name to the Asiatic specimen (*Cuming 119*, Luzon, Philippines) but as Smith’s the name is validated by Heward’s description it is typified by the Heward specimen from Jamaica.

#### Description.

Rhizome erect, rising at least 20 cm above ground, diameter 1.5 cm. Rhizome scales pseudopeltate to peltate, 4–7 × 1.0–1.7 mm, narrowly lanceolate, attenuate toward apex. Fronds 100–130 × 40–50 cm; stipes slender, 37–40 cm long, 0.5 –1 cm across (at base), dark brown; lamina deltoid, tripinnate, 70–100 × 40–50 cm, rather thick herbaceous, rigid, brown-green when dry, scaly; pinnae at 40–45° to rachis, largest at base, slightly overlapping, stalked 1 cm, including stalk up to 23–25 × 9–10 cm, lanceolate, first basiscopic pinnules of lowest pinnae enlarged; ultimate segments 0.9–2.0 × 0.3–0.6 cm, sessile or very short-stalked, oblong trapeziform, apex obtuse to acute, margin with weak cartilaginous border; distance from vein to base of sinuses ca. 1.0 mm; crenate-dentate, shallowly to deeply incised, lobes obtuse to acute, veins in lobes 3–15 forks, light to dark brown, not strongly contrasting with lamina, percurrent. Scales on rachis mostly present at base of pinnae or pinnules, irregularly triangular, basifix, 0.7–0.9 × 0.3–0.4 mm.

#### Distribution.

Sumatra, Peninsular Malaysia (Pahang, Perak, Selangor), Java, Borneo (Sabah, Kalimantan Timur), Philippines (Leyte, Luzon, Mindanao, Negros), Moluccas (Seram), New Guinea, Vanuatu (Aneityum), Fiji (Vanua Levu, Taveuni) (Figure 8).

#### Ecology.

Terrestrial, mostly in shaded moist habitats, river banks, moist slopes, ravines etc., in primary or disturbed forest, at 0–2000 m altitude.

#### Discussion.

*Orthiopteris campylura* is the most variable species within the genus. It is widely distributed from Western Malesia to Papua New Guinea and the Pacific, existing as different entities in each sub-geographical region, which in many cases have been recognized as individual species. However, we have regularly observed samples that were intermediate between the forms, and accordingly treat them here as varieties rather than as subspecies. Characters of the sori provide the best criteria to distinguish these varieties.

There are no data that suggest that any of the varieties is distinguished by a different ecology.

### a. Orthiopteris campylura var. campylura

Taxon classificationPlantaePolypodialesSaccolomataceae

Figs 3a, b , 6d

#### Description.

Sori protruding from the margin on distinct lobes, in one plane with lamina wings, 0.9–1 × 0.5–0.8 mm, symmetric, narrowly funnelform-obovate, widest at mouth; inner indusium brown, firm, equal to outer indusium, sometimes longer, apex similar to outer indusium; outer indusium truncate or obtuse, shallowly undulate; sporangia 7–12 per sorus, capsule globose and rounded at apex, gradually narrowed toward base, indurated annulus cells 15–20, clearly unequal; spores in polar view 35–45 µm, in lateral view 30–33 µm.

#### Distribution.

Philippines (Leyte, Luzon, Mindanao, Negros), Moluccas (Seram), Papua New Guinea (Manus).

#### Discussion.

Although in the shape of inner and outer indusium shape this species resembles Orthiopteris campylura var. kingii, the sori are not reflexed but usually flat even when dried. The distribution center of this variety appears to be in the Philippines.

### b. Orthiopteris campylura var. caudata

Taxon classificationPlantaePolypodialesSaccolomataceae

(Copel.) P.H.Hovenkamp & T.T.Luong comb. nov.

urn:lsid:ipni.org:names:77148378-1

Figs 2e , 3g

Saccoloma caudatum Copel. Philipp. J. Sci. 30: 327. 1926.
Ithycaulon caudatum (Copel.) Copel., Univ. Calif. Publ. Bot. 16: 80. 1929b. Type. Based on *Saccoloma caudatum* Copel.
Orthiopteris caudata (Copel.) Copel. Philipp. J. Sci. 78: 9. 1950. Type. Based on *Saccoloma caudatum* Copel.

#### Type.

PAPUA NEW GUINEA. Hydrographers Range, alt. 900 m, *King 462* (holo: MICH, 1190968* [http://quod.lib.umich.edu/h/herb2ic/x-mich1190968/mich1190968.tif]).

#### Description.

Sori not protruding from the margin on distinct lobes, not reflexed when dry, in one plane with lamina wings, 0.7–0.9 × 0.6–0.7 mm, asymmetric, funnelform, widest at mouth of sori; inner indusium bright-brown, firm, usually shorter but not shorter than 2/3 length of outer indusium, apex with c. 0.2 mm long lobe (< 1/3 length of sorus); outer indusium emarginate to undulate-truncate; sporangia 17–21 per sorus, capsule globose and rounded at apex, gradually narrowed toward base, indurated annulus cells 17–20, clearly unequal; spores in polar view 28–32 µm, in lateral view 25–28 µm.

#### Distribution.

North Moluccas, Papua New Guinea.

### c. Orthiopteris campylura var. kingii

Taxon classificationPlantaePolypodialesSaccolomataceae

(Bedd.) P.H.Hovenkamp & T.T.Luong comb. nov.

urn:lsid:ipni.org:names:77148379-1

Figs 2g , 3c , 4d, e, f

Dicksonia kingii Bedd., Handb. Suppl.: 6. 1892.
Dennstaedtia acuminata Rosenst., Hedwigia 56: 350. 1915. Type. PAPUA NEW GUINEA. Sattelberg, alt. 800 m, April 1914. *G. Bamler 139* (holo: B, B200044968* [http://herbarium.bgbm.org/object/B200044968]; iso: P, P00633321* [http://coldb.mnhn.fr/catalognumber/mnhn/p/p00633321], P00633320* [http://coldb.mnhn.fr/catalognumber/mnhn/p/p00633320]; UC, 391869* [http://plants.jstor.org/stable/10.5555/al.ap.specimen.uc391869]; S, S-P-6347* [http://andor.nrm.se/kryptos/fbo/kryptobase/large/S-P-006001/S-P-6347.jpg], S09-41598* [http://andor.nrm.se/kryptos/fbo/kryptobase/large/S09-041001/S09-41598.jpg]).
Ithycaulon acuminatus (Rosenst.) Copel., Univ. Calif. Publ. Bot. 12: 395. 1931. Type. Based on *Dennstaedtia acuminata* Rosenst.
Orthiopteris acuminata (Rosenst.) Copel. Philipp. J. Sci. 78: 9. 1950. Type. Based on *Dennstaedtia acuminata* Rosenst.
Orthiopteris kingii (Bedd.) Holttum, Rev. Fl. Mal. 2: 306. f. 175. 1954. Type. Based on *Dicksonia kingii* Bedd.
Saccoloma kingii (Bedd.) Parris in Parris, R.S. Beaman & Beaman, Pl. Mt. Kinabalu I. Ferns & Fern Allies: 151. 1992; G.B.Nair, J. Econ. Taxon. Bot. 16 (3): 645. “1992” [1994]. Type. Based on *Dicksonia kingii* Bedd.

#### Type.

PENINSULAR MALAYSIA. Gunong Batu Puteh, August 1885, *King’s collectors 8058* (holo: K, 2 sheets: K000794854 [http://specimens.kew.org/herbarium/K000794854]; K000794855 [http://specimens.kew.org/herbarium/K000794855]; iso: SING, 0170905).

#### Description.

Sori protruding from the margin, strongly reflexed when dry, in one plane with lamina wings, 0.9–1.0 × 0.5–0.8 mm, symmetric, narrowly funnelform-obovate, widest just below mouth; inner indusium dark-brown, firm, shorter or equal to outer indusium, apex similar to outer indusium; outer indusium truncate or obtuse, shallowly undulate tip; sporangia 6–12 per sorus, capsule globose and rounded at apex, gradually narrowed toward base, indurated annulus cells 17–20, clearly unequal; spores in polar view 28–30 µm, in lateral view 20–22 µm.

#### Distribution.

Sumatra, Peninsular Malaysia (Pahang, Perak, Selangor), Java, Borneo (Sabah, Kalimantan Timur), Moluccas (Seram), Papua New Guinea.

#### Discussion.

This variety is widespread in western Malesia. It is easily recognized when dry by its distinctly protruding and reflexed sori. The ultimate segments of this variety may be the widest among the varieties of *Orthiopteris campylura*, although many collections from the Moluccas have narrow segments. The abaxial surface is scaly, but the scales are very small and not easily observed.

### d. Orthiopteris campylura var. insularis

Taxon classificationPlantaePolypodialesSaccolomataceae

P.H.Hovenkamp & T.T.Luong var. nov.

urn:lsid:ipni.org:names:77148376-1

Figs 2i , 3i, j

#### Diagnosis.

Sori asymmetric, not protruding from the margin, < 0.6 mm wide. outer indusium at one side strongly excurrent on lamina.

#### Type.

SOLOMON ISLANDS. San Cristoval, Hinuahaoro, alt. 900 m, 22 September 1932, *Brass 2914* (holo: L, 0319881, iso: K, MICH).

#### Description.

Sori not protruding from the margin, not reflexed, in one plane with lamina wings, 0.8–1.0 × 0.5–0.6 mm, asymmetric, narrow obovate, widest just below mouth sori; inner indusium bright-brown, firm, usually shorter, sometimes equal to outer indusium, apex with 0.1-0.2 mm long lobe; outer indusium deeply emarginate, at one side strongly excurrent on lamina; sporangia 10–15 per sorus, capsule globose and rounded at apex, gradually narrowed toward base, indurated annulus cells 17–22, clearly unequal; spores not seen.

#### Distribution.

Papua New Guinea (Bougainville), Solomon islands, Vanuatu (Erromango).

#### Etymology.

The varietal epithet refers to the archipelagic distribution of this taxon.

#### Discussion.

This variety is quite widespread in the island archipelago stretching from Bougainville to the New Hebrides, but appears to be absent on the mainland of New Guinea.

### e. Orthiopteris campylura var. laxa

Taxon classificationPlantaePolypodialesSaccolomataceae

P.H.Hovenkamp & T.T.Luong var. nov.

urn:lsid:ipni.org:names:77148377-1

Figs 2f , 3o, p

#### Diagnosis.

Lobes of ultimate segments blunt. Sori symmetric, outer indusium truncate, not confluent with lamina margin on one side but incised.

#### Type.

FIJI. Vanua Levu, Taveuni, 29 December 1933, *A.C. Smith 865* (holo: NY).

#### Description.

Lamina not complete for measurement of size and shape, firm-herbaceous, yellowish-green, scales not found; ultimate segments 1.4–1.5 × 0.6–0.7 cm, sessile or very short-stalked, trapeziform, base asymmetric, cuneate, apex obtuse to acute, margin shallowly incised, distance from base of sinuses to costules 0.7–1.5 mm, lobes blunt; veins in lobes 3–15 forks, light to dark brown, not strongly contrasting with lamina, percurrent.

Ultimate segments 1.4–1.5 × 0.6–0.7 cm, sessile or very short stalked, trapeziform, Sori protruding from margin, not reflexed, 0.9–1.0 × 0.8 mm, symmetric, narrowly funnelform, widest at mouth; inner indusium brownish, firm, slightly shorter than outer indusium, apex with 0.2–0.3 mm long lobe, 1/4–1/3 length of sorus; outer indusium truncate, emarginate or undulate; sporangia 10–15 per sorus, capsule globose and rounded at apex, gradually narrowed toward base, indurated annulus cells 17–22, unequal in size; spores not seen.

#### Distribution.

Vanuatu (Aneityum), Fiji (Vanua Levu, Taveuni).

#### Etymology.

The varietal epithet refers to the general aspect of the fronds.

#### Discussion.

The specimens grouped here have been confused with *Orthiopteris firma* mainly because of the similarity in the lobed inner indusium, but differ from that species in sorus shape and a less distinct cartilaginous border. The two available specimens examined here also show a considerable variation among themselves. More specimens or molecular evidence are needed to provide better support for the existence of this new variety.

### 2. Orthiopteris cicutarioides

Taxon classificationPlantaePolypodialesSaccolomataceae

(Baker) Copel., Univ. Calif. Publ. Bot. 18: 217. 1942.

Figs 2b , 3h , 4a

Davallia cicutarioides Baker, J. Bot. 28: 106 (1890)
Leucostegia ? cicutarioides (Baker) C.Chr., Index Filic., Suppl. III: 120 (1934) Type. Based on *Davallia cicutarioides* Baker
Ithycaulon tenuisectum C.Chr., Brittonia 2: 285. 1937. Type. PAPUA NEW GUINEA. Central Division, Dieni, alt. 700 m, 01 May 1933, *Brass 3139* (holo: BM n.v., iso: NY, 00127937*; MICH, 1190678*)
Ithycaulon cicutariodes (Baker) Alston, J. Bot. 77: 289. 1939. Type. Based on *Davallia cicutarioides* Baker

#### Type.

PAPUA NEW GUINEA. Mount Musgrave, alt. 700 m, 25 June 1889, *W. Macgregor s.n.* (Holo: K, 00794858*).

#### Description.

Rhizome erect, rising at least 2 cm above ground, diameter unknown. Rhizome scales pseudopeltate, ca. 3.0 × 1.0 mm, lanceolate with long falcate acumen. Fronds 90–110 × 30-50 cm; stipes stout, 35–45 cm long, 0.4–0.5 cm across (at base), light brown; lamina deltoid, tripinnate to quadripinnate, ca. 70 × 30–50 cm, papyraceous, yellow-green when dry, glabrous or with few scattered scales; pinnae at 40–45° to rachis, largest at base, overlapping, stalk 2.0–2.5 cm, including stalk 25–35 × 10–15 cm, lanceolate, first basiscopic pinnules of lowest pinnae enlarged; ultimate segments 1.0–2.0 × 0.5–0.7 cm, sessile or very short stalked, trapeziform, apex acute, margin with weak cartilaginous border; deeply incised to less than 0.2 mm from veins; lobes oblanceolate, veins in lobes unbranched or with 1 fork, light brown and not strongly contrasting with lamina, percurrent. Scales on rachis mostly present at base of segment, hair-like. Sori apical on small lobes, lateral on larger lobes, symmetric, not reflexed, slightly concave with lamina wings slightly recurved, 0.7–0.8 × 0.4–0.5 mm, wide obovate, widest just below mouth, not wider than the lamina below sorus; inner indusium brownish, firm, shorter than outer indusium, apex with c. 0.1 mm long lobe; outer indusium truncate-obtuse.

#### Distribution.

Papua New Guinea (Boridi).

#### Ecology.

Terrestrial, at 700–1500 m altitude.

#### Discussion.

*Orthiopteris cicutarioides* has the least dissected fronds in the group of finely dissected-frond species, with usually more than one vein per lobe. The single specimen of this species that could be examined had sterile sori without sporangia, which might be an indication of a hybrid origin. The shape of the sorus and indusium of *Orthiopteris cicutarioides* resembles those of Orthiopteris campylura var. caudata, and it could represent a locally derived taxon.

### 3. Orthiopteris ferulacea

Taxon classificationPlantaePolypodialesSaccolomataceae

(Moore) Copel., Bernice P. Bishop Mus. Bull. 59: 14. 1929a.

Figs 2a , 3l , 4b

Davallia trichomanoides Hook., Sec. cent. Ferns T 64. 1861, *non*Blume 1828.
Davallia ferulacea T. Moore, Index Fil. (T. Moore): 294. 1861. Type. Based on *Davallia ferulacea* T. Moore
Saccoloma ferulaceum (T. Moore) R.M.Tryon & A.F.Tryon, Rhodora 84: 127. 1982. 1982a. Type. Based on *Davallia ferulacea* T. Moore
Saccoloma ferulaceum (T. Moore) G.B.Nair, J. Econ. Taxon. Bot. 16(3): 642. “1992” [1994]. Type. Based on *Davallia ferulacea* T. Moore

#### Type.

FIJI. Naviti Levu, Dec. 1855, *Milne 315* (holo: K, 2 sheets: K00794861*, K00794862*).

#### Description.

Rhizome erect, rising at least 2–3 cm above ground, diameter 5–7 mm. Rhizome scales peltate or pseudopeltate, 2.5–3.0 × 0.7–0.9 mm, lanceolate with long falcate acumen. Fronds 30–80 × 10–30 cm; stipes slender, 10–30 cm long, 0.1–0.2 cm across (at base), light brown; lamina deltoid, tripinnate to quadripinnate, 20–50 cm × 10–30 cm, papyraceous, bright green when dry, glabrous; pinnae at 30–35° to rachis, largest at base, overlapping, stalk 0.5 cm, including stalk 15–25 × 6–9 cm, lanceolate, first basiscopic pinnules of the lowest pinnae enlarged; ultimate segments 1–2 × 0.5 cm, sessile or very short stalked, trapeziform, apex acute, margin without or with very weak cartilaginous border; deeply incised to less than 0.1 mm from veins; lobes narrowly oblanceolate, veins in lobes unbranched, light brown and not strongly contrasting with lamina, ending well below apex. Scales on rachis mostly at base of pinnae or pinnule, irregular triangular, basifixed. Sori apical on lobes, symmetric, not reflexed when dried, in one plane with lamina wings, 0.7–1.0 × 0.4–0.5 mm, elliptic, widest at middle or slightly above, wider than the sterile lamina below sorus; inner indusium brownish green, thin and slightly transparent, usually shorter, sometimes equal to outer indusium, apex with narrow 0.2–0.3 mm long tongue, c. 1/2 length of sorus; outer indusium obtuse; sporangia 4–7 per sorus, capsule globose and rounded at apex, gradually narrowed toward base, indurated annulus cells 16–18, ±equal; spores in polar view 26–30 µm, in lateral view 20–25 µm.

#### Distribution.

endemic to Fiji (Viti Levu).

#### Ecology.

Terrestrial in dense forest, bank of creek, at 50–1200 m altitude.

#### Discussion.

*Orthiopteris ferulacea* is the smallest species in the genus and the most easily recognizable among the species group with finely dissected fronds because of its relative small size and delicate and finely dissected frond. *Orthiopteris trichophylla*, similarly dissected, has larger fronds and funnel-shaped sori without narrow tongue, whereas sori of *Orthiopteris ferulacea* are elliptic with a narrow tongue.

### 4. Orthiopteris firma

Taxon classificationPlantaePolypodialesSaccolomataceae

(Kuhn) Brownlie, Fl. Nouv.-Calédonie & Dépend. 3: 112, t. 13, f. 9, 10. 1969.

Figs 3m, n , 4c , 5b , 6a, b

Saccoloma moluccanum var. firmum Kuhn, Verh. Zool.-Bot. Ges. Wien 19: 582. 1869.
Saccoloma firmum (Kuhn) C.Chr., Vierteljahrsschr. Naturf. Ges. Zürich 70. 221. 1925. Type. Based on var. firmum Kuhn
Ithycaulon firmum (Kuhn) C.Chr., Index Filic., Suppl. III: 116. 1934. Type. Based on var. firmum Kuhn

#### Lectotype

**(here designated).** VANUATU. Erromango, *MacGillivray s.n* (B, 200157508 [http://herbarium.bgbm.org/object/B200157508]).

#### Description.

Rhizome erect, rising at least 20 cm above ground, diameter unknown (complete rhizome not seen). Rhizome scales basifixed, round to lanceolate, round scales 0.5–0.85 × 0.6–1.0 mm, cordate scales 1.2–1.5 × 1.1–1.5 mm, lanceolate scales 0.7–0.8 × 0.–2.5 mm. Fronds ca 100–150 cm long; stipes stout, of unknown length, ca. 0.7–0.8 cm across (at base), dark brown; lamina deltoid, quadripinnate, complete fronds not seen, herbaceous, thick, dark green when dry, glabrous; pinnae at 50–60° to rachis, largest at base, separated or slightly overlapping, stalk 3 cm, including stalk up to 40 × 25 cm, lanceolate, first basiscopic pinnules of lowest pinnae enlarged; ultimate segments 2.0–3.0 × 0.7–1.5 cm, sessile or very short stalked, narrow trapeziform, apex obtuse to acute, margin with distinct, thick, yellow cartilaginous border; shallowly incised to 1.0–2.0 mm from veins; lobes acute, veins in lobes 7–15 forks, bright green, clearly visible and contrasting with lamina, percurrent, joining with border. Scales on rachis only on rachis or veins, very sparse (few scales on an entire frond), irregular triangular, basifixed, 0.35–0.50 × 0.25–0.4 mm. Sori apical on small lobes, lateral on larger lobes, symmetric, not or slightly reflexed, slightly concave with lamina wings usually recurved, 1.4–1.2 × 0.8 mm, elliptic to narrowly obovate, widest at middle to 3/4 from base; inner indusium bright brown and contrasting in colour with lamina, firm, slightly shorter than outer indusium, sometimes equal, apex with narrow 0.35-0.45 mm long tongue, 1/3–1/2 length of sorus; outer indusium acute, obtuse, rarely truncate or emarginate; sporangia 9–15 per sorus, capsule rectangular-triangular with truncate apex, narrowed toward base, indurated annulus cells 17–20, unequal; spores in polar view 30–32 µm, in lateral view 25–27 µm.

#### Distribution.

New Caledonia; New Hebrides (?), Fiji (?)

#### Ecology.

Terrestrial on slopes or margin of humid montane forest, at 300–900 m altitude.

#### Discussion.

This species is quite large and therefore often incompletely represented in collections. Rhizomes are usually missing and entire fronds are not preserved. The brightly colored, firm and large sori are very conspicuous on the dark green lamina. The majority of the specimens of *Orthiopteris firma* are from New Caledonia, except for the two specimens cited in the protologue and a single specimen from Fiji (*Betche s.n.*). All these represent records more than 100 years old for each respective location (collecting date is neither mentioned in literature nor indicated on specimen labels but collectors lived before 1913). For at least one of these specimens, a mistake in labelling is likely, as a similar specimen (*MacGillivray s.n.* B 200157509) is indeed labelled as originating from New Caledonia. All recent collections of *Orthiopteris firma* are from New Caledonia, and therefore *Orthiopteris firma* is best considered as an endemic for New Caledonia.

Kuhn (1869) described Saccoloma moluccanum var. firma based on two specimens: *Herus 85* (Aneityum, New Hebrides) and *MacGillivray s.n.* (Erromango, New Hebrides, B200157508). Our study concludes that they belong to different taxa. *Herus 85* is Orthiopteris campylura var. laxa, *MacGillivray s.n*. is this taxon, although probably with an erroneous location, and is here selected as the lectotype for Saccoloma moluccanum var. firmum to maintain nomenclatural stability.

### 5. Orthiopteris henriettae

Taxon classificationPlantaePolypodialesSaccolomataceae

(Baker) Copel., Gen. Fil.: 50. 1947.

Figs 2h , 3q, r, s , 5a , 6f

Dicksonia henriettae Baker in Hook. & Baker, Syn. Fil. (ed. 2): 462. 1874.
Microlepia henriettae (Baker) Kuhn, Reisen Ost-Afrika (Decken): 62. 1879. Type. Based on *Dicksonia henriettae* Baker
Dennstaedtia henriettae (Baker) Diels, Nat. Pflanzenfam. 1: 4: 218. 1899. Type. Based on *Dicksonia henriettae* Baker
Saccoloma henriettae (Baker) C.Chr., Dansk Botanisk Arkiv 7: 75, t. 25, f. 12–13. 1932. Type. Based on *Dicksonia henriettae* Baker
Ithycaulon henriettae (Baker) C.Chr. Index Filic., Suppl. III: 116. 1934. Type. Based on *Dicksonia henriettae* Baker

#### Type.

MADAGASCAR. Antananarivo, *Baker s.n*. (holo: K, 000351013* [http://specimens.kew.org/herbarium/K000351013]).

#### Description.

Rhizome erect, rising at least 5 cm above ground, diameter 1.5–2 cm. Rhizome scales pseudopeltate–peltate, 1.5–3.0 × 0.5–1.3 mm, narrow lanceolate with long sinuose acumen, thick. Fronds 110–150 × 100–150 cm (length fairly equal with width); stipes slender, 30–50 cm long, 0.4–0.6 cm across (at base), dark brown; lamina rhomboid–deltoid, widest at 3–5 cm above the base, tripinnate to quadripinnate (lowest lobe of ultimate segments free or nearly so), 60–110 × 140–150 cm, papyraceous, thin, dull brown–yellowish green when dry, glabrous; pinnae at 45–50° to rachis, largest at the second from base, overlapping, stalk 3 cm, including stalk 30–50 × 10–15 cm, lanceolate, first acrosopic, sometimes first basiscopic pinnule of the lowest pinnae enlarged; ultimate segments 3–5 × 1.5–2.5 cm, 1–2 mm stalked, ovate-trapeziform, with strongly asymmetric base, lowest lobe almost completely separated, apex obtuse, margin with weak cartilaginous border; shallowly incised to 1.5–2.5 mm from veins; lobes rounded, veins in lobes 8–9 forks, dark brown, not contrasting with lamina, percurrent. Scales on rachis a few hair-like scales only on veins (few scales on an entire frond), hair-like. Sori lateral on lobes, symmetric, not reflexed, sometimes concave with lamina wings recurved, 0.8–1.1 × 0.5–1.0 mm, wide funnelform-obovate, sometimes narrow funnelform, widest at mouth or just below mouth; inner indusium dull brown, papyraceous, thin but tough, with swollen joint at base with distinct dark line at the joint, 1/2 – 2/3 as long as outer indusium, apex emarginate and irregularly eroded, not extending into a lobe; outer indusium truncate to obtuse; sporangia 10–17 per sorus, capsule globose and rounded at apex, gradually narrow edtoward base, indurated annulus cells 18–24, unequal in size; spores in polar view 30–38 µm, in lateral view 22–25 µm.

#### Distribution.

endemic to Madagascar (Toamasina, Antsiranana, Toliara, Tamatave, Antananarivo).

#### Ecology.

Terrestrial in moist, shady, evergreen forest, on laterite soil derived from gneiss or in lowland dense disturbed forest with bamboo and Acanthaceae, border of the creek etc., at 350–1700 m altitude.

#### Discussion.

*Orthiopteris henriettae* is the only representative of the genus in Madagascar, where it is not rare. It is clearly distinct from the Malay-Pacific species by its fronds having a papyraceous and thin texture, the ovate-trapeziform ultimate segments with shallowly crenate-dentate margin and usually swollen joints in the veins just below sori. The ultimate segments seem to be narrower in collections from higher altitudes.

### 6. Orthiopteris samoensis

Taxon classificationPlantaePolypodialesSaccolomataceae

P.H.Hovenkamp & T.T.Luong spec. nov.

urn:lsid:ipni.org:names:77148375-1

Figs 2c , 3t , 3u, v , 4g, h , 6g, h

#### Diagnosis.

Differs from all other *Orthiopteris* species in relatively dense scales on lower surface of the lamina.

#### Type.

SAMOA. Savai’i, 31 December 1905, *Vaupel 312* (holo: L, 3 sheets: L0319870, L0319927, L0319847, iso: MO, U).

#### Description.

Rhizome erect, rising at least 50 cm above ground, diameter unknown. Rhizome scales unknown. Fronds 150–200 cm (complete fronds not seen); stipes stout, ca. 70 cm long, 0.5–0.8 cm across (at base), light brown; lamina probably deltoid, quadripinnate, 100–150 cm long, at least 60 cm wide, firm- herbaceous, brownish green when dry, scaly with scattered scales on abaxial surface, denser at base of segments, and on veins and base of rachis; pinnae at 45–50° to rachis, separated or slightly overlapping, stalk 1.6 cm, including stalk up to 30–45 × 15–20 cm, lanceolate; ultimate segments 1.0–2.0 × 0.4-0.8 cm, sessile or very short stalked, narrowly trapezoid, apex obtuse to acute, margin with weak cartilaginous border; shallowly incised to 0.3-1.0 mm from veins; lobes obtuse to acute, veins in lobes form 3–6 forks, yellow green, weakly contrasting with lamina, percurrent. Scales on rachis irregularly triagular, basifixed, 0.4–1.4 × 0.4–0.9 mm. Sori lateral on lobes, symmetric, not reflexed, in one plane with lamina wings, 0.5–0.8 × 0.6–0.75 mm, suborbicular to funnelform, widest at mouth; inner indusium brown to dark brown, firm, slightly shorter or equal to outer indusium, rarely longer, apex with a rounded to truncate, 0.1–0.2 mm long lobe, eroded, ca. ¼ length of sorus; outer indusium truncate, shallowly lobed or eroded; sporangia 5–10 per sorus, capsule globose and rounded at apex, gradually narrowed toward base, indurated annulus cells 17–19, clearly unequal; spores in polar view 30–33 µm, in lateral view 23–27 µm.

#### Distribution.

endemic to Samoa (Upolu, Savaii).

#### Ecology.

Terrestrial, medium wet forest, at 600–900m altitude

#### Etymology.

The specific epithet refers to the type locality.

#### Discussion.

The key character to separate this newly recognised species from the others are the many scales covering the abaxial surface of lamina. *Orthiopteris campylura* may have some scales on the lower lamina surface, but these are sparser, smaller and narrower. Apart from the laminal scales, *Orthiopteris samoensis* most closely resembles Orthiopteris campylura var. caudata in terms of frond and sori shape, but the inner indusia of *Orthiopteris samoensis* are not lobed.

### 7. Orthiopteris tenuis

Taxon classificationPlantaePolypodialesSaccolomataceae

(Brack.) Brownlie, Nova Hedwigia 55 (Pterid. Fl. Fiji): 115. 1977.

Figs 3e, f, k

Microlepia tenuis Brack., U.S. Expl. Exped., Filic. 16: 236. 1854.
Microlepia papillosa Brack. U.S. Expl. Exped., Filic. 16: 237, t. 34, fig. 1. 1854. Type. FIJI. *U.S South Pacific Exploring Expedition s.n.* (number 4 in Brackenridge 1854) (Holo: US, 00134882*[http://plants.jstor.org/stable/10.5555/al.ap.specimen.us00134882]; iso: K, 000794859*).
Saccoloma tenue (Brack.) Mett., Ann. Sci. Nat., Bot. sér. 4, 15: 80. 1861. Type. Based on *Microlepia tenuis* Brack.
Saccoloma papillosa (Brack.) Mett., Ann. Sci. Nat., Bot. sér. 4, 15: 80. 1861. Type. Based on *Microlepia papillosa* Brack.

#### Type.

FIJI. *U.S South Pacific Exploring Expedition s.n.* (number 3 in Brackenridge 1854) (Holo: US, 00134883* [http://plants.jstor.org/stable/10.5555/al.ap.specimen.us00134883]; iso: K & NY, K000794860*, NY 00127936*).

#### Description.

Rhizome erect, rising at 5–60 cm above ground, diameter 1.5–10 cm. Rhizome scales pseudopeltate, 4–8 × 0.7–1 mm, narrow, linear lanceolate, usually falcate and suddenly contracted into a long thin acumen. Fronds 100–170 × 40–50 cm; stipes slender, 30–70 cm long, 0.3–0.8 cm across (at base), dark brown; lamina deltoid, widest at base, tripinnate, sometimes quadripinnate in large plants, ca. 70 × 40 cm, herbaceous, lively green when dry, glabrous; pinnae at 35–45° to rachis, largest at base, separated or slightly overlapping, stalk 1–2 cm, including stalk up to 26 × 12 cm, lanceolate, first basiscopic pinnules of lowest pinnae enlarged; ultimate segments 1.5–2.0 × 0.7–1.0 cm, sessile or very short stalked, trapezoid to narrowly so near frond apex, apex obtuse to acute or attenuate, margin with weak cartilaginous border; shallowly to deeply incised to 0.2–2.0 mm (see discussion) from veins; lobes acute, veins in lobes with 1–3 forks, bright green, strongly contrasting to the lamina, percurrent, sometimes ending just below apex. Scales on rachis absent, absent. Sori apical on small lobes, lateral on larger lobes, asymmetric, sometimes symmetric, not reflexed, in one plane with lamina wings, ca. 1.5 × 1 mm, funnelform, sometimes ovate, widest at middle to 2/3 from base; inner indusium yellow bright green, contrasting in colour with lamina, firm, 1/2–2/3 as long as outer indusium, apex with obtuse to acute lobe, slightly eroded, ca. 0.25–0.5 the length of inner indusium; outer indusium obtuse, sometimes acute, truncate or emarginate with 1–2 shallow sinuses; sporangia 7–10 per sorus, capsule globose and rounded at apex, gradually narrowed toward base, indurated annulus cells 17–22, ±equal; spores in polar view 30–35 µm, in lateral view 25–27 µm.

#### Distribution.

Endemic to Fiji (Viti Levu, Vanua Levu, Ovalau).

#### Ecology.

Terrestrial, dense forest, bank along stream, at 0–1000 m altitude.

#### Discussion.

This species is highly variable in terms of frond dissection, and sorus shape. Plants from higher altitudes (above ca. 500 m) have larger fronds and furthermore differ from the lowland plants in ultimate segments being deeply incised (distance of lamina from base of sinuses to costules less than 0.5 mm), and sori with almost equally long inner- and outer indusium. In contrast, the lowland plants have ultimate segments more shallowly incised (distance of lamina from base of sinuses to costules more than 0.5 mm), and sori with a large difference in length between inner and outer indusia. We could not separate the two forms because of the presence of intermediate specimens.

### 8. Orthiopteris trichophylla

Taxon classificationPlantaePolypodialesSaccolomataceae

Copel.

Figs 2d , 3d

Orthiopteris trichophylla Copel., Univ. Calif. Publ. Bot. 18: 217. 1942.
Saccoloma trichophyllum (Copel.) G.B.Nair, J. Econ. Taxon. Bot. 16(3): 643. “1992” [1994]. Type. Based on *Orthiopteris trichophylla* Copel.

#### Type.

PAPUA NEW GUINEA. Idenburg River, 15 km southwest of Bernhard camp, alt.1800 m, 1939, *Brass 12027*, (erroneously cited as *Brass 12057* in Copeland 1942, holo: MICH, 1190791* [http://quod.lib.umich.edu/h/herb2ic/x-mich1190791/mich1190791.tif]; iso: BM, 001044444, BO 1510941)

#### Description.

Rhizome erect. Rhizome scales not seen. Fronds 120–160 × 40–50 cm; stipes stout, 40–50 cm long, 0.4–0.6 cm across (at base), dark brown; lamina deltoid, widest at base, tripinnate to quadripinnate, 100–120 × 40–50 cm, herbaceous, dark brownish green when dry, glabrous or with few scattered scales; pinnae at 30–35° to rachis, largest at base, overlapping, stalk 0.5 cm, including stalk 15–25 × 6–9 cm, lanceolate, first basiscopic pinnules of lowest pinnae enlarged; ultimate segments 1.0–1.5 × 0.5 cm, sessile or very short stalked, trapezoid, apex acute, margin with weak cartilaginous border; almost completely dissected to less than 0.2 mm from veins; lobes narrowly oblong, veins in lobes unbranched or with 1 fork, dark brown and not contrasting with lamina, percurrent. Scales on rachis mostly at base of pinnae or pinnule, very few on veins, hair-like. Sori lateral or apical on lobes, symmetric, slightly reflexed, slightly concave with lamina wings slightly recurved, 1.0–1.2 × 0.6–0.7 mm, funnelform, widest at mouth, not wider than the sterile lamina below sorus; inner indusium dull brown, firm, 4/5–2/3 as long as outer indusium, apex truncate; outer indusium truncate; sporangia 7–12 per sorus, capsule globose and rounded at apex, gradually narrowed toward base, indurated annulus cells 16–20, ±equal; spores in polar view not studied, in lateral view.

#### Distribution.

Papua New Guinea (Idenburg River).

**Figure 9.**
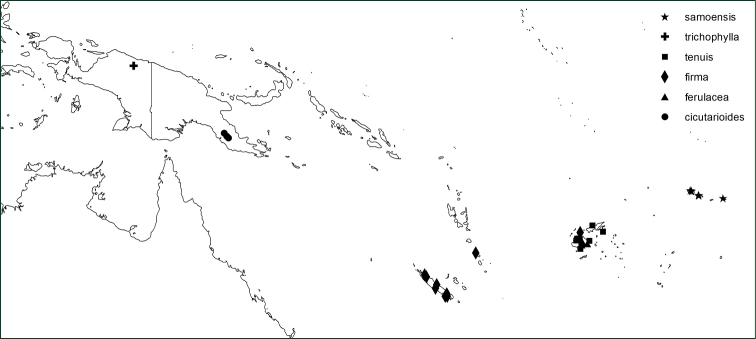
Distribution of *Orthiopteris* species in East Malesia and the Pacific.

#### Ecology.

Terrestrial in bottom of ravines, rain forest, at 1800 m altitude.

#### Discussion.

This species can be mistaken for *Orthiopteris cicutarioides* because the frond morphology is similar, with fronds very finely dissected in both species. However, *Orthiopteris trichophylla* is more finely dissected with a single vein per lobe (similar to *Orthiopteris ferulacea*), while *Orthiopteris cicutarioides* has 2 or 3 one veins per lobe. The sori and indusia of *Orthiopteris trichophylla* resemble those of Orthiopteris campylura var. kingii, being funnelform and slightly reflexed, and the possibility cannot be excluded that *Orthiopteris trichophylla* is locally derived from Orthiopteris campylura var. kingii. The single specimen we examined (*Brass 12239*) differs slightly from the type (studied from scanned image of *Brass 12027*) by its shorter lobes. However, the sori and indusia are identical, and they were collected on the same day and at the same locality. Unlike in *Orthiopteris trichophylla*, which may represent a similar case of a locally derived taxon, both specimens of *Orthiopteris cicutarioides* look very productive with many ripe sporangia spreading over the mouth of the sorus. Brass noted that the species was very common and abundant at the collecting site (Idenburg river), but we have not seen any later collections than Brass’s in 1939. Perhaps it is just abundant locally, and it may well be currently threatened.

### Excluded names

***Ithycaulon* Copel., Univ. Calif. Publ. Bot. 16: 79. 1929b.**

Type. *Ithycaulon moluccanum* (Blume) Copel.

*Ithycaulon moluccanum* (Blume) Copel, Univ. Calif. Publ. Bot. 16: 79. 1929b.

= *Saccoloma moluccanum* (Blume) Mett in Kuhn, Verh. zool. bot. Ges. 19. 581. 1869.

Both names *Ithycaulon moluccanum* and *Saccoloma moluccanum* are based on *Davallia moluccana* Blume, Enum. Pl. Javae 2: 237 (1828), for which the accepted name is now *Tapeinidium moluccanum* (Blume) C.Chr, Gard. Bull. Straits Settlem. 4: 399. (1929). For more details of this misapplication see De Joncheere (1984).

## Supplementary Material

XML Treatment for Orthiopteris

XML Treatment for Orthiopteris campylura

XML Treatment for Orthiopteris campylura var. campylura

XML Treatment for Orthiopteris campylura var. caudata

XML Treatment for Orthiopteris campylura var. kingii

XML Treatment for Orthiopteris campylura var. insularis

XML Treatment for Orthiopteris campylura var. laxa

XML Treatment for Orthiopteris cicutarioides

XML Treatment for Orthiopteris ferulacea

XML Treatment for Orthiopteris firma

XML Treatment for Orthiopteris henriettae

XML Treatment for Orthiopteris samoensis

XML Treatment for Orthiopteris tenuis

XML Treatment for Orthiopteris trichophylla

## Appendix

Identification list Orthiopteris

1a. Orthiopteris campylura var. campylura

1b. Orthiopteris campylura var. caudata

1c. Orthiopteris campylura var. kingi

1d. Orthiopteris campylura var. insularis

1e. Orthiopteris campylura var. laxa

2. *Orthiopteris cicutarioides*

3. *Orthiopteris ferulacea*

4. *Orthiopteris firma*

5. *Orthiopteris henriettae*

6. *Orthiopteris samoensis*

7. *Orthiopteris tenuis*

8. *Orthiopteris trichophylla*

Alston, AHG 16940: 1b;

anon. 1123: 6; 119: 6; 249: 6; s.n.: 6;

Aridy, J; Rahajasoa, G; Moise 69: 5;

Baker s.n.: 5;

Bamler, MG ROS 116: 1b;

Baudouin 1805?: 4;

Baumann-Bodenheim, MG 10168: 4; 10236: 4; 15190: 4;

Baumann-Bodenheim, MG; Guillaumin, A 10281: 4;

Beer’s collectors BSIP 7671: 1d;

Betche, E s.n.: 4; s.n.: 6;

Boerlage, JG 687: 1c; 687: 1c;

Braithwaite, AF RSNH 2268: 1d; RSS 4110: 1d; RSS 4245: 1d;

Brass, LJ 2914: 1d; 3050: 1d; 3919: 2; 12027: 8; 12239: 8; 12269: 1c; 12941: 1c;

Brownlie, G 771: 3;

Carr, CE 12518: 1b; 13067: 1b; 13257: 2;

Chew, WL; Corner, EJH; Stainton, A 1578: 1c;

Christophersen, E; Hume, EP 2032: 6; 2065: 6;

Clemens, J; Clemens, MS 40386: 1c;

Clemens, MS 3470: 1c; 5120: 1c;

Copeland, EB 21777: 1a; PPE 67: 1a;

Croft, JR 799: 1b; 1206: 1a; 1720: 1c; 1959: 1b; 2053: 1b;

Cuming, H 119: 1a; 360: 1a;

Curtis, C 2085: 1c;

Degener, O 14648: 7; 14907: 3; 15162: 7;

Dorr, LJ 3144: 5;

Edaño, GE BS 78693: 1a; BS 79627: 1a; PNH 807: 1a; PNH 11183: 1a; PNH 11187: 1a; PNH 11223: 1a; PNH 17313: 1a;

Elmer, ADE 9067: 1a; 9714: 1a; 10183: 1a; 11717: 1a;

Franc (BONATI) 36: 4; (BONATI) 335: 4; (ROSENSTOCK) 4: 4;

Gay, H 404: 1c;

Germain, R s.n.: 4;

Gillespie, JW 3326: 3; 4013: 7; 5124: 3;

Green, PS 1793: 4;

Grether, DF; Wagner, WH 4161: 1a;

Guillaumin, A; Baumann-Bodenheim, MG 10318: 4;

Herus 85: 1e;

Hildebrandt, JM 3765: 5;

Holttum, RE 11477: 1c; 15412: 1c; 31220: 1c;

Hoogland, RD; Craven, LA 10162: 1c; 10386: 1c;

Hovenkamp, PH; Arifiandy, NM; Iqbal, M 05/ 159: 1a;

Humbert, H 14131: 5; 22461: 5; 23155: 5;

Humbert, H; Capuron, R; Cours, G 24686: 5;

Humbert, H; Cours, G 17864: 5;

Iqbal, M; Usman 049: 1a;

Iwatsuki, K; Kato, M; Ueda, K; Mahjar, UW C 302: 1c; C 1025: 1c;

Janssen, T 2418: 5;

John, H 18311: 3;

Kajewski, SF 2672: 1d;

Kato, M C 13703: 1c;

Kato, M; Imaichi, R; Aso, K; Darnaedi, D; Ujang Hapid B 860: 1a;

Kato, M; Okamoto, M; Walujo, EB B 9266: 1c; B 9584: 1c; B 10082: 1c; B 11308: 1c; B 11498: 1c;

Kato, M; Okamoto, M; Walujo, EB; Ueda, K; Darnaedi, D B 8363: 1c;

Kato, M; Sunarno, B; Akiyama, H C 3224: 1c;

Kato, M; Ueda, K; Fanani, Z C 11298: 1c; C 11350: 1c; C 11642: 1c; C 11683: 1c; C 12934: 1c; C 13231: 1c; C 13330: 1c; C 14106: 1c;

Kato, M; Ueda, K; Mahjar, UW C 1248: 1c; C 1294: 1c; C 1771: 1c; C 2121: 1c; C 2149: 1c; C 2160: 1c;

Kato, M; Ueda, K; Mahjar, UW; Okamoto, M; Akiyama, H; Sunarno, B C 5372: 1c; C 5613: 1c; C 6548: 1c; C 6876: 1c; C 7159: 1c; C 7478: 1c;

King’s collector 2118: 1c; 8058: 1c;

Koroiveibau, D; Saula 16119: 3;

Kuhl, H; Hasselt, JC van s.n.: 1c;

Lam, HJ 7222: 4;

Lam, HJ; Meeuse, ADJ 5841: 5; 5892: 5;

Loher, A s.n.: 1a; Copel. 6716: 1a;

Lörzing, JA 5787: 1c; 6910: 1c; 14722: 1c;

MacGillivray, J s.n.: 4;

Main s.n.: 1b;

Malcomber, S; Rakotomalaza, PJ; Raharilala, J 2246: 5;

Matthews, CG 649: 1c; s.n.: 1c;

McGregor, RC 24: 2;

McPherson, G 6528: 4;

Merrill, ED 5951: 1a;

Miller, JS 3545: 5;

Milner s.n. (313): 3; s.n. (315): 3;

Mohd Haniff 9081: 1c; 9083: 1c;

Morrison, A s.n.: 1e;

Mueller, F von s.n.: 1e;

Murton, HJ 26: 1c;

Nakaike, T 108: 1c;

Palmer, W; Bryant, O 547: 1c;

Pancher, M s.n.: 4;

Parks, HE 20050: 7; 20192: 7; 20861: 7; 212332: 3;

Parris, BS; Croxall, JP 7910: 1c;

Powell, T s.n.: 6;

Price, MG 2478: 1a; 3166: 1a;

Pullen, R 3453: 1b; 7889: 1c; 7924: 1c; 8263: 1b; 8265: 1b;

Raciborski, M 126: 1c;

Rakotondrainibe, F 3163: 5;

Rakotovao, C; Felix, E; Razafindasy, R; Antilahimena, P; Razanatsoa, H 1233: 5;

Ramos, M BS 14795: 1a; BS 30424: 1a; BS 39545: 1a;

Randriambololomamonjy, O; Razakamalala, R; Jaowind 347: 5;

Ravololonanahary, H; Ralimanana, H; Birkinshaw, C; Randrianaivo, R; Ranaivoj 44: 5;

Razafitsalama, LJ 486: 5;

Reinecke 149 b: 6; 97 a: 6;

Reinecke, F 71: 6;

Ridley, HN 8634: 1c; 14000: 1c;

Ridsdale, CE; Lavarack, P NGF 30654: 1b; NGF 30656: 1d;

Robinson, HC s.n.: 1c;

Sands, MJS; Pattison, GA; Wood, JJ 2924: 1a;

Schlechter, R 14890: 4;

Schodde, R 1562: 1c;

Scortechini, B 306: 1c;

Seemann, B 753: 7;

Shim, PS San 81811: 1c;

Sledge, WA 1563: 6;

Smith, AC 4381: 7; 6463: 7; 6764: 7; 7274: 7; 8533: 7; 865: 7; 8875: 7; 8885: 3; 8932: 3; 9154: 7;

Stevens, PF; Lelean, Y LAE 58676: 1b;

Sulit, MD PNH 20294: 1a;

Surbeck, H 756: 1c; 1085: 1c; 1175: 1c;

Tabualewa, A 15613 a: 3; 15566: 7;

Turnau, EA 936: 1c;

U.S South Pacific Exploring Expedition s.n. 3: 7; s.n. 4: 7;

Vaupel, F 312: 6; 482: 6;

Venning, FEW MA 18: 1c;

Vieillard, E 1623: 4;

Wenzel, CA 646: 1a;

Werff, HH van der; McPherson, G 15906: 4;

Werff, HH van der; McPherson, G; Rapanarivo, S 13622: 5;

Whitmore, TC BSIP 1572: 1d;

Whitmore, TC; Grubb, PJ BSIP 2067: 1d;

Wiriadinata, H 1457: 1c;

Yapp, RH 427: 1c;

Zollinger, H 244: 1c;
